# Identifies KCTD5 as a novel cancer biomarker associated with programmed cell death and chemotherapy drug sensitivity

**DOI:** 10.1186/s12885-023-10895-2

**Published:** 2023-05-06

**Authors:** Yuan-Xiang Shi, Jian-Hua Yan, Wen Liu, Jun Deng

**Affiliations:** 1 Institute of Clinical Medicine, Hunan Provincial People’s Hospital, The First Affiliated Hospital of Hunan Normal University, Changsha, Hunan 410005 P.R. China; 2 Department of Cardiac Thoracic Surgery, Hunan Provincial People’s Hospital, The First Affiliated Hospital of Hunan Normal University, Changsha, Hunan China; 3 Department of Pharmacy, Hunan Provincial People’s Hospital, The First Affiliated Hospital of Hunan Normal University, Changsha, Hunan 410005 P.R. China

**Keywords:** KCTD5, Prognosis, Tumor microenvironment, Cell apoptosis, Drug sensitivity, Pan-cancer

## Abstract

**Background:**

More and more studies have demonstrated that potassium channel tetramerization domain-containing 5 (KCTD5) plays an important role in the development of cancer, but there is a lack of comprehensive research on the biological function of this protein in pan-cancer. This study systematically analyzed the expression landscape of KCTD5 in terms of its correlations with tumor prognosis, the immune microenvironment, programmed cell death, and drug sensitivity.

**Methods:**

We investigated a number of databases, including TCGA, GEPIA2, HPA, TISIDB, PrognoScan, GSCA, CellMiner, and TIMER2.0. The study evaluated the expression of KCTD5 in human tumors, as well as its prognostic value and its association with genomic alterations, the immune microenvironment, tumor-associated fibroblasts, functional enrichment analysis, and anticancer drug sensitivity. Real-time quantitative PCR and flow cytometry analysis were performed to determine the biological functions of KCTD5 in lung adenocarcinoma cells.

**Results:**

The results indicated that KCTD5 is highly expressed in most cancers and that its expression is significantly correlated with tumor prognosis. Moreover, KCTD5 expression was related to the immune microenvironment, infiltration by cancer-associated fibroblasts, and the expression of immune-related genes. Functional enrichment analysis revealed that KCTD5 is associated with apoptosis, necroptosis, and other types of programmed cell death. In vitro experiments showed that knockdown of KCTD5 promoted apoptosis of A549 cells. Correlation analysis confirmed that KCTD5 was positively correlated with the expression of the anti-apoptotic genes Bcl-xL and Mcl-1. Additionally, KCTD5 was significantly associated with sensitivity to multiple antitumor drugs.

**Conclusion:**

Our results suggest that KCTD5 is a potential molecular biomarker that can be used to predict patient prognosis, immunoreactions and drug sensitivity in pan-cancer. KCTD5 plays an important role in regulating programmed cell death, especially apoptosis.

**Supplementary Information:**

The online version contains supplementary material available at 10.1186/s12885-023-10895-2.

## Introduction

Human health is seriously threatened by cancer, a major public health problem worldwide. According to GLOBOCAN 2020, the numbers of cancer cases and cancer deaths worldwide in 2020 were 19,292,789 and 9,958,133, respectively [1]. The top three causes of death due to cancer in the United States in 2022 were lung, prostate and colorectal cancer in men and lung, breast and colorectal cancer in women [2]. With the progress of precision medicine and the use of targeted drugs, the level of cancer treatment has been effectively improved, but drug resistance and tumor metastasis remain major challenges. At present, research is still focused on identifying cancer-related prognostic markers and therapeutic targets.

Potassium channel tetramerization domain-containing (KCTD) proteins contain a conserved N-terminal domain and a variable C-terminal domain. Members of this family of proteins contain a common BTB domain at the N-terminus that is involved in transcriptional repression, regulation of the cytoskeleton, and binding to the cullin E3 ubiquitin ligase complex [3]. Previous studies have reported that KCTD family proteins are related to cancer, neurological disease, and metabolic disorders through the regulation of the Hedgehog, Wnt/beta-catenin, GABA and other signaling pathways [3–6]. In our previous studies, KCTD proteins have been implicated in the regulation of hypoxic microenvironments and immune invasion in lung adenocarcinoma (LUAD) [7]. Potassium channel tetramerization domain-containing 5 (KCTD5) is an important member of the KCTD protein family that has been proposed as a putative substrate-specific adaptor for cullin3-based E3 ligases [8]. The function of KCTD5 in cancer development has been shown in increasing numbers of studies, but no comprehensive studies of its role in pan-cancer have been conducted.

This study systematically investigated the expression landscape of KCTD5 across cancers through multiple databases. Importantly, the relationships between KCTD5 expression and genomic mutations, prognosis, the immune microenvironment, cancer-associated fibroblasts (CAFs), and drug sensitivity were revealed. We also performed pathway enrichment analysis and found that KCTD5 is associated with programmed cell death. Furthermore, we confirmed the involvement of KCTD5 in the regulation of lung cancer cell apoptosis in vitro.

## Materials and methods

### Data collection and comprehensive analysis

UCSC Xena provides gene expression data, clinicopathologic data, survival data, and genetic variation data on 33 cancers that appear in TCGA (Table 1) [9]. We used the R package “UCSCXenaShiny” to study the differential expression of KCTD5 in pan-cancer. We also measured the expression of KCTD5 across cancers in the GEPIA2 database (http://gepia2.cancer-pku.cn/) [10]. The HPA database (https://www.proteinatlas.org/) was used to investigate the expression of KCTD5 in a variety of tumor cell lines [11]. The UALCAN database (https://ualcan.path.uab.edu/) was used to evaluate the protein expression of KCTD5 in pan-cancer. The TISIDB database (http://cis.hku.hk/TISIDB/) was used to demonstrate the distribution of KCTD5 expression across immune and molecular subtypes [12]. To analyze the effect of KCTD5 expression on tumor patient prognosis, the Kaplan–Meier Plotter was used (http://kmplot.com/analysis/) [13]. We also assessed the relationship between KCTD5 expression and survival time in pan-cancer using PrognoScan (http://dna00.bio.kyutech.ac.jp/PrognoScan/index.html) [14]. The prognostic indicators used were overall survival (OS), disease-free survival (DFS), disease-specific survival (DSS), distant metastasis-free survival (DMFS), and relapse-free survival (RFS). The GSCA database (http://bioinfo.life.hust.edu.cn/GSCA/#/) was used to analyze copy number variation (CNV), single nucleotide variation (SNV), methylation and drug sensitivity of KCTD5 in pan-cancer and their associations with gene expression [15]. The CellMiner database (https://discover.nci.nih.gov/cellminer/) was used to evaluate the relationship between KCTD5 expression and drug sensitivity. The immune landscape of KCTD5 was analyzed using the TIMER2.0 (http://timer.cistrome.org/) and TISIDB databases [16]. The LinkedOmics database (http://linkedomics.org/login.php) contains multiomics data from all cancer types represented in TCGA and in 10 CPTAC cancer cohorts [17]. In this study, the LinkedOmics database was used to perform KEGG enrichment analysis of KCTD5 in pan-cancer [18]. The statistical method was the Pearson correlation test. FDR < 0.05 was considered statistically significant.

**Table 1:** List of cancer types

Abbreviation	Cancer Types
ACC	Adrenocortical carcinoma
AML	Acute myeloid leukemia
BLCA	Bladder urotelial carcinoma
BRCA	Breast invasive carcinoma
CESC	Cervical squamous cell carcinoma and endocervical adenocarcinoma
CHOL	Cholangiocarcinoma
COAD	Colorectal adenocarcinoma
DLBC	Lymphoid Neoplasm Diffuse Large B-cell Lymphoma
ESCA	Esophageal carcinoma
GBM	Glioblastoma multiforme
HNSC	Head and neck squamous cell carcinoma
KICH	Kidney Chromophobe carcinoma
KIRC	Kidney renal clear cell carcinoma
KIRP	Kidney renal papillary cell carcinoma
LGG	Brain Lower Grade Glioma
LIHC	Liver hepatocellular carcinoma
LUAD	Lung adenocarcinoma
LUSC	Lung squamous cell carcinoma
MESO	Mesothelioma
OV	Ovarian serous cystadenocarcinoma
PAAD	Pancreas adenocarcinoma
PCPG	Pheochromocytoma and paraganglioma
PRAD	Prostate adenocarcinoma
READ	Rectal adenocarcinoma
SARC	Sarcoma
SKCM	Skin cutaneous melanoma
STAD	Stomach adenocarcinoma
TGCT	Testicular Germ Cell Tumors
THCA	Thyroid carcinoma
THYM	Thymoma
UCEC	Uterine Corpus Endometrial Carcinoma
UCS	Uterine Carcinosarcoma
UVM	Uveal Melanoma

### Cell culture and transfection

The human normal lung epithelial cell line BEAS-2B and the human lung cancer cell lines A549, H292, H1299 were purchased from the Chinese Academy of Sciences and cultured at 37 °C in 5% CO_2_. The lung cancer cell lines were cultured in RPMI-1640 medium containing 10% fetal bovine serum (FBS), and BEAS-2B cells were cultured in DMEM containing 10% FBS. KCTD5-targeted siRNA, siRNA-KCTD5 and negative control scrambled siRNA (SCR) were synthesized by Guangzhou RiboBio Company. The final concentration of siRNA was 50 nM, and cells were collected 48 h after transfection for measurement of gene expression and apoptosis.

### RNA extraction and real-time quantitative PCR

First, total RNA was extracted with TRIzol® reagent (Invitrogen; Thermo Fisher Scientific Inc.). Reverse transcription was performed using the PrimeScript™ RT reagent kit (Takara Bio, Inc.), and RT‑qPCR was performed using the SYBR Green PCR kit (Takara Bio, Inc.), both according to the protocols provided by the manufacturer. The primer sequences used were as follows: GAPDH forward, 5'‑CCCATCACCATCTTCCAGGAG‑3' and reverse, 5'‑GTTGTCATGGATGACCTTGGC‑3'; KCTD5 forward, 5'‑TCCAAGTGGGTCCGACTCAA‑3' and reverse, 5'‑GGTCTCTGTCGATTAAATAGGCG‑3'. GAPDH was used an internal reference gene, and data were calculated using the 2^−∆∆CT^ method [19]. *P* < 0.05 was considered statistically significant.

### Flow cytometry analysis

The Annexin V‑PE/7-AAD Apoptosis Kit (cat. no. AP104, MULTISCIENCES Inc.) was used to detect cell apoptosis. We used flow cytometry (CytoFLEX Flow Cytometer, Beckman Coulter, Inc.) to analyze apoptosis. *P* < 0.05 was considered statistically significant.

## Results

### Expression and mutational landscape of KCTD5 in pan-cancer

**Fig. 1:**
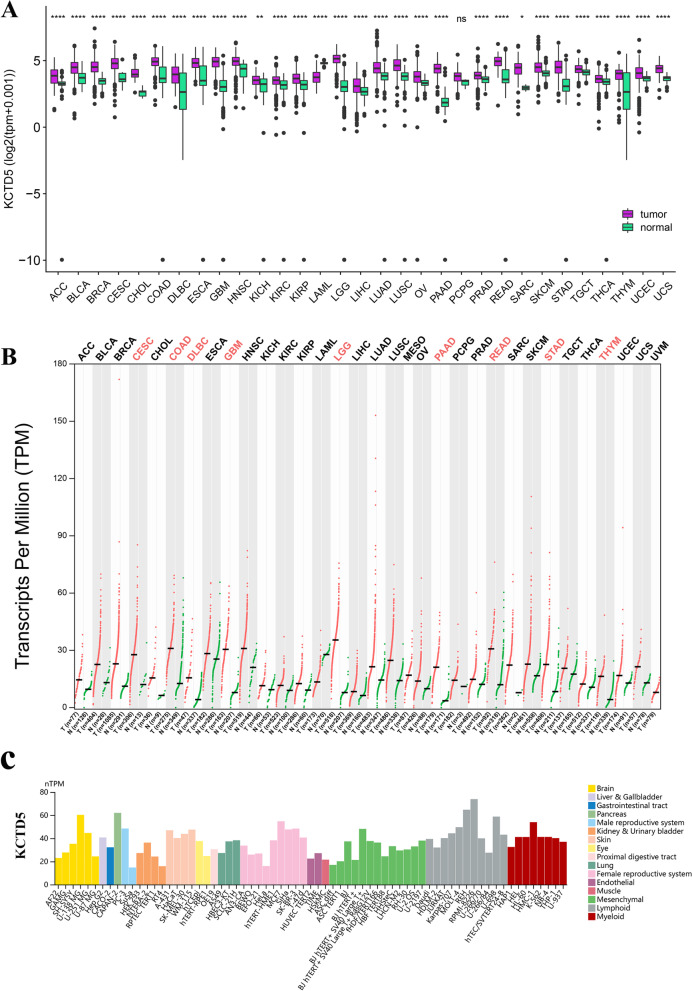
The expression landscape of KCTD5 in pan-cancer. **A** The expression level of KCTD5 in tumor tissues and adjacent normal tissues of 33 TCGA cancer from TCGA datasets. **B** The expression level of KCTD5 in different tumor tissues and corresponding normal tissues from TCGA and GTEx datasets. **C** The expression levels of KCTD5 in multiple tumor cell lines (HPA dataset). *P < 0.05; **P < 0.01; ***P < 0.001

To identify the pattern of expression of KCTD5 in tumors, we measured KCTD5 expression in tissue samples (TCGA database) and cells (the HPA database). The results showed that KCTD5 is expressed at significantly higher levels in most tumors (ACC, BLCA, BRCA, CESC, CHOL, COAD, DLBC, ESCA, GBM, HNSC, KICH, KIRC, KIRP, LGG, LIHC, LUAD, LUSC, OV, PAAD, PRAD, READ, SARC, SKCM, STAD, TGCT, THCA, THYM, UCEC) than in normal tissues, with the exception of LAML and PCPG (Fig. 1A). Subsequent expression analysis using the GEPIA2 database confirmed the above results (Fig. 1B). Figure 1C shows that KCTD5 is highly expressed a variety of tumor cells, including the brain cancer cell lines AF22, GAMG, SH-SY5Y, U-138 MG, U-251MG, and U-87 MG, the kidney and urinary bladder cancer cell lines HEK 293, NTERA-2, and RT4, the lung cancer cell lines A549, HBEC3-KT, and SCLC-21H, and in female reproductive system cancer cells such as AN3-CA, BEWO, EFO-21, HeLa, hTERT-HME1, MCF7, SiHa, SK-BR-3, and T-47d. We also evaluated the protein expression level of KCTD5 in pan-cancer (UALCAN database). The results showed that KCTD5 was highly expressed in glioblastoma (*P* < 0.001), head and neck squamous carcinoma (*P* < 0.001), hepatocellular carcinoma (*P* < 0.001), lung adenocarcinoma (*P* < 0.001), pancreatic adenocarcinoma (*P* < 0.001), clear cell RCC (*P* < 0.001), and uterine corpus endometrial carcinoma (UCEC) (*P* < 0.05) (Supplementary Fig. 1).

**Fig. 2:**
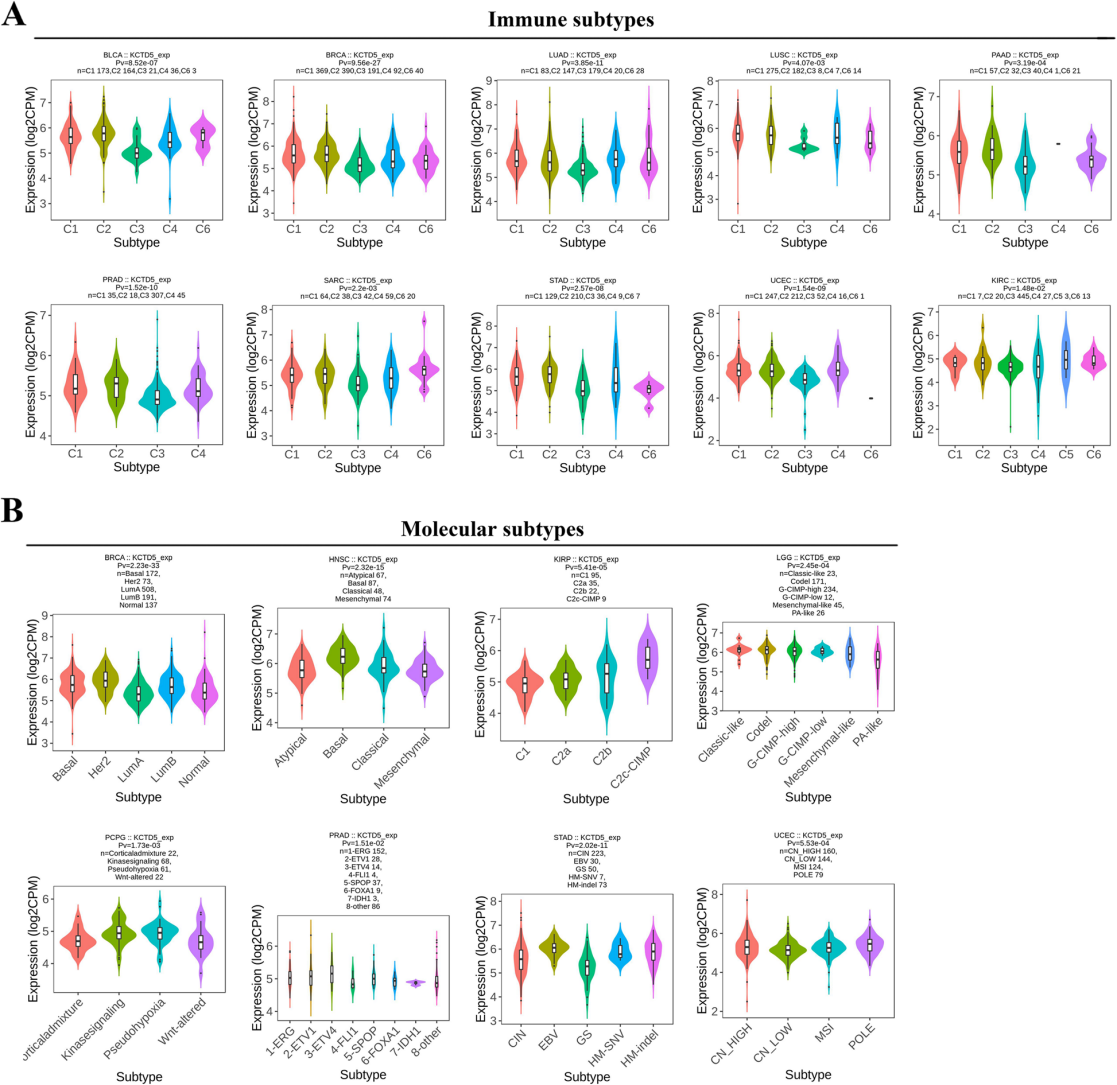
The correlation between KCTD5 expression and immune subtypes and molecular subtypes in pan-cancer. **A** Expression levels of KCTD5 in different immune subtypes. C1 (wound healing); C2 (IFN-gamma dominant); C3 (inflammatory); C4 (lymphocyte depleted); C5 (immunologically quiet); C6 (TGF-b dominant). **B** Expression levels of KCTD5 in different molecular subtypes

Furthermore, we measured the expression of KCTD5 in cells with different immune and molecular subtypes in pan-cancer (Fig. 2). The violin plots show that KCTD5 expression is significantly correlated with immune and molecular subtype in multiple tumors. KCTD5 expression showed significant correlation with immune subtypes in BLCA (*P* = 8.52e-07), BRCA (*P* = 9.56e-27), LUAD (*P* = 3.85e-11), LUSC (*P* = 4.07e-03), PAAD (*P* = 3.19e-04), PRAD (*P* = 1.52e-10), SARC (*P* = 2.2e-03), STAD (*P* = 2.57e-08), UCEC (*P* = 1.54e-09), and KIRC (*P* = 1.48e-02). KCTD5 was expressed at a low level in the C3 subtype in all 10 cancers examined. The expression of KCTD5 in molecular subtypes of BRCA (*P* = 2.23e-33), HNSC (*P* = 2.32e-15), KIRP (*P* = 5.41e-05), LGG (*P* = 2.45e-04), PCPG (*P* = 1.73e-03), PRAD (*P* = 1.51e-02), STAD (*P* = 2.02e-11), and UCEC (*P* = 5.53e-04) was all significantly different. KCTD5 was highly expressed in the Her2 subtype of BRCA and the basal subtype of HNSC. It was found that high levels of KCTD5 were expressed in the C2c-CIMP subtype of KIRP, whereas low levels of KCTD5 were found in the C1 subtype.

**Fig. 3:**
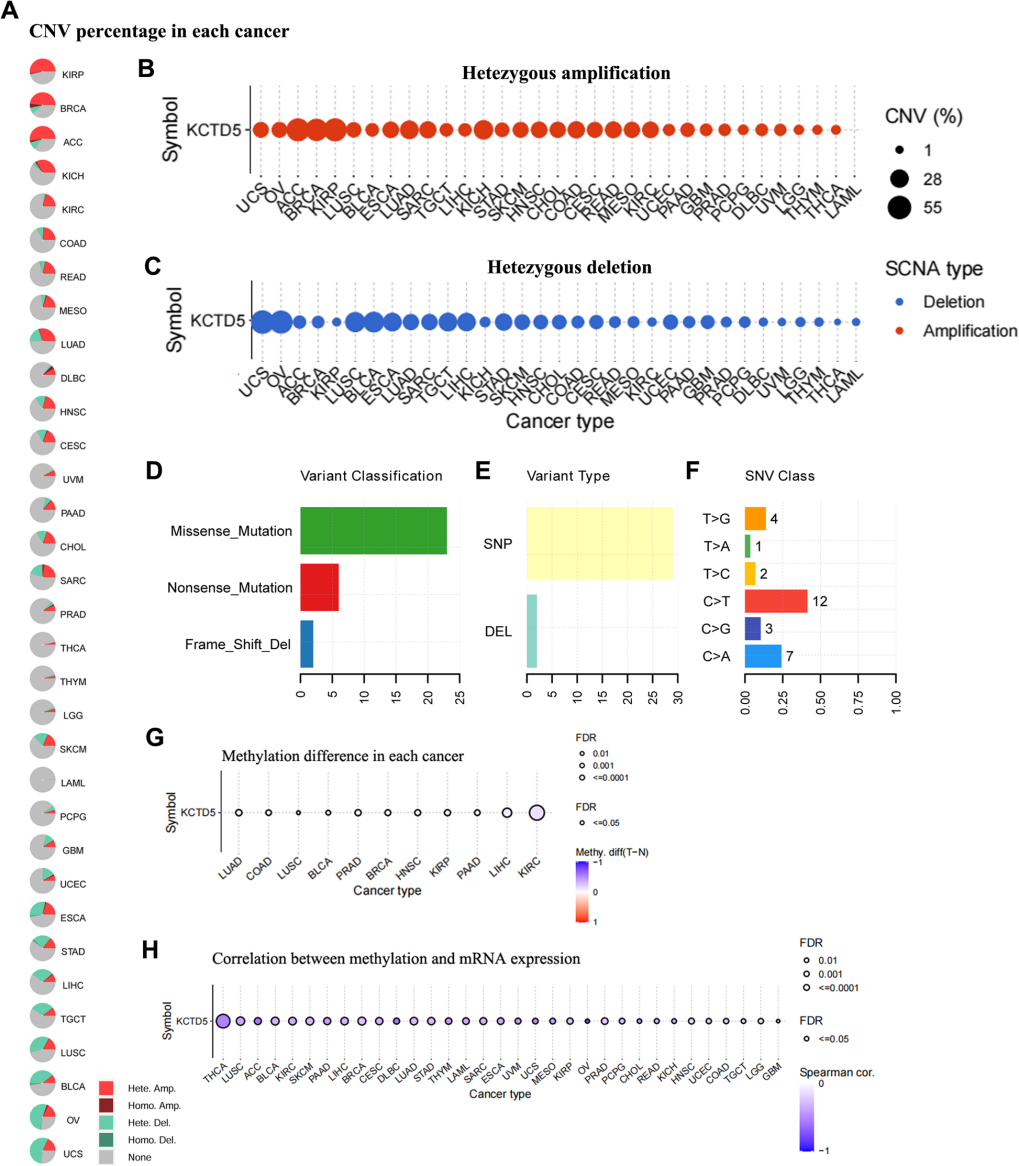
The genetic alterations and DNA methylation of KCTD5 across cancers. **A-C** The change frequency of CNV in KCTD5 was revealed by GSCA database. Different colors in the pie chart represent different types of variation. Hete Amp (heterozygous amplification): CNV = 1; Hete Del (heterozygous deletion): CNV = -1. **D-F** The SNV classes of KCTD5 in pan-cancer. **G** The differential methylation analysis of KCTD5 in a variety of tumors. **H** The correlation analysis showed that the methylation level of KCTD5 was negatively correlated with mRNA expression

Genetic alterations and DNA methylation of the KCTD5 gene across cancers were analyzed using the GSCA database. The results showed that CNV of KCTD5 occurred frequently in KIRP, BRCA, ACC, KICH, LUAD, SARC, ESCA, LUSC, BLCA, OV and UCS, while in LAML, LGG, THYM, and THCA, its frequency was low (Fig. 3A). In addition, we investigated heterozygous amplifications (CNV = 1) and heterozygous deletions (CNV = -1) of KCTD5 in various cancers and found that heterozygous amplifications occurred frequently in KIRP, BRCA, ACC, and KICH, while heterozygous deletions were more common in UCS, OV, BLCA, and LUSC (Fig. 3B-C). Next, we analyzed SNV across cancers and found that missense mutations occurred most frequently in KCTD5 (Fig. 3D-F). The methylation analysis suggested that there were significant differences in the methylation levels of KCTD5 in KIRC, LIHC, LUAD, COAD, LUSC, BLCA, PRAD, BRCA, HNSC, KIRP, and PAAD (FDR < 0.05) (Fig. 3G). Importantly, association analysis showed a negative correlation between KCTD5 methylation and its expression level in multiple tumors (Fig. 3H). The above results demonstrate that there is highly heterogeneous genetic variation in KCTD5 across different cancers.

### Prognostic value of KCTD5

**Fig. 4:**
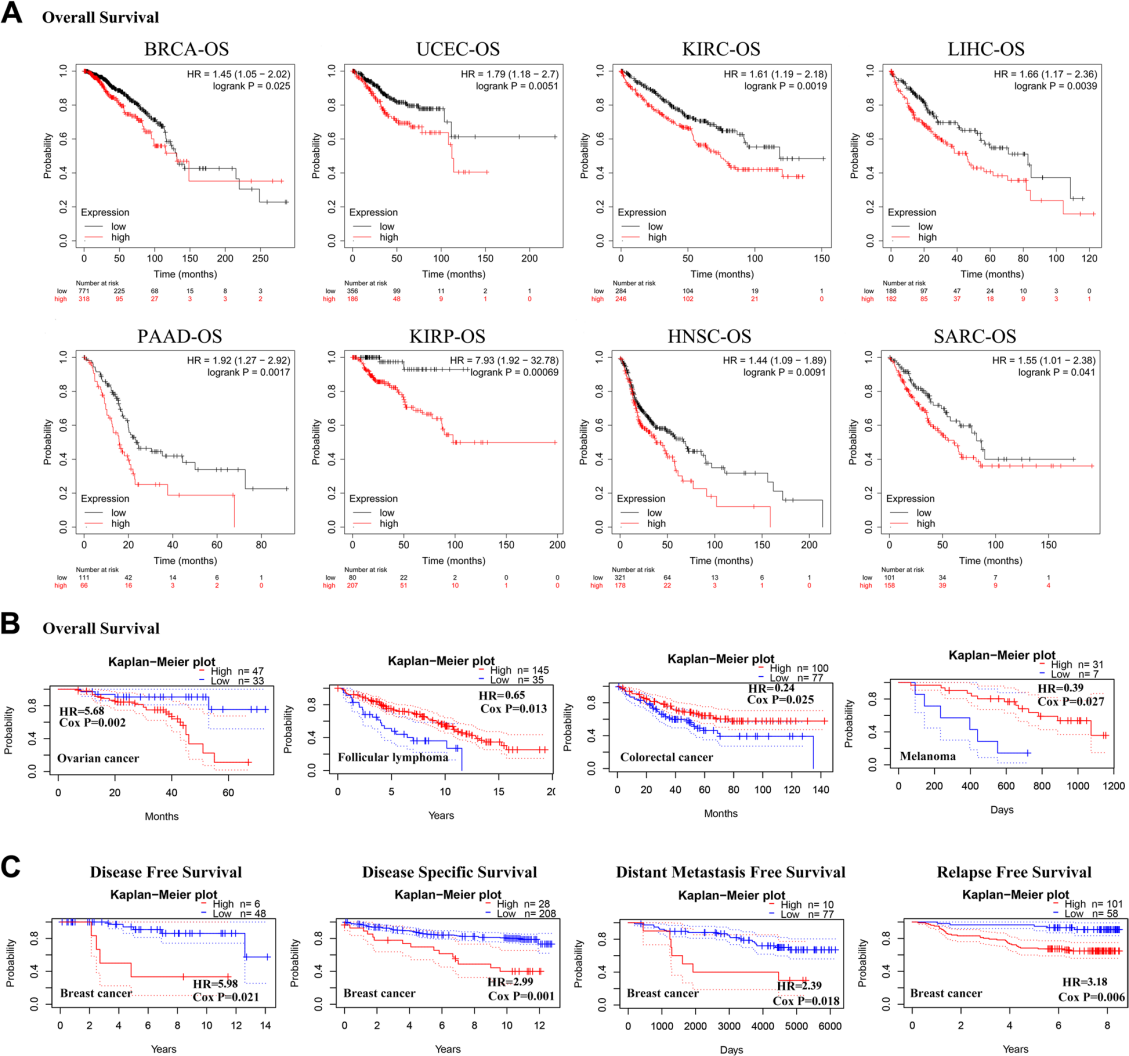
Survival analysis of KCTD5 in pan-cancer. We assessed the prognostic value of KCTD5 in pan-cancer using the Kaplan–Meier plotter (**A**) and the PrognoScan (**B**, **C**) database. **A**, **B** Overall survival (OS) was used as an indicator. **C** The survival analysis including disease-free survival (DFS), disease-specific survival (DSS), distant metastasis-free survival (DMFS), and relapse-free survival (RFS) of KCTD5 in breast cancer

We assessed the prognostic value of KCTD5 in pan-cancer using the Kaplan–Meier plotter and the PrognoScan database. The OS, DFS, DSS, DMFS and RFS of the patients were used as indicators. The Kaplan–Meier prognostic analysis indicated that there was a statistically significant difference in OS between the groups with high and low expression of KCTD5 in BRCA (*P* = 0.025, HR = 1.45), UCEC (*P* = 0.005, HR = 1.79), KIRC (*P* = 0.002, HR = 1.61), LIHC (*P* = 0.004, HR = 1.66), PAAD (*P* = 0.002, HR = 1.92), KIRP (*P* = 0.001, HR = 7.93), HNSC (*P* = 0.009, HR = 1.44) and SARC (*P* = 0.041, HR = 1.55) (Fig. 4A). Among patients with these types of tumors, OS was shorter in the group with high expression of KCTD5 (Fig. 4A). The PrognoScan survival analysis also revealed significant differences in OS between the high and low expression groups of KCTD5 in ovarian cancer (*P* = 0.002, HR = 5.68), follicular lymphoma (*P* = 0.013, HR = 0.65), colorectal cancer (*P* = 0.025, HR = 0.24), and melanoma (*P* = 0.027, HR = 0.39) (Fig. 4B). We found that ovarian cancer patients with high expression of KCTD5 had poor prognoses (Fig. 4B). In patients with breast cancer, there were significant differences in DFS (*P* = 0.021, HR = 5.98), DSS (*P* = 0.001, HR = 2.99), DMFS (*P* = 0.018, HR = 2.39) and RFS (*P* = 0.006, HR = 3.18) between the high-expression KCTD5 group and the low-expression KCTD5 group, suggesting that the high expression of KCTD5 is associated with poor prognosis in breast cancer (Fig. 4C).

### Immune aspects of KCTD5 in the tumor microenvironment

**Fig. 5:**
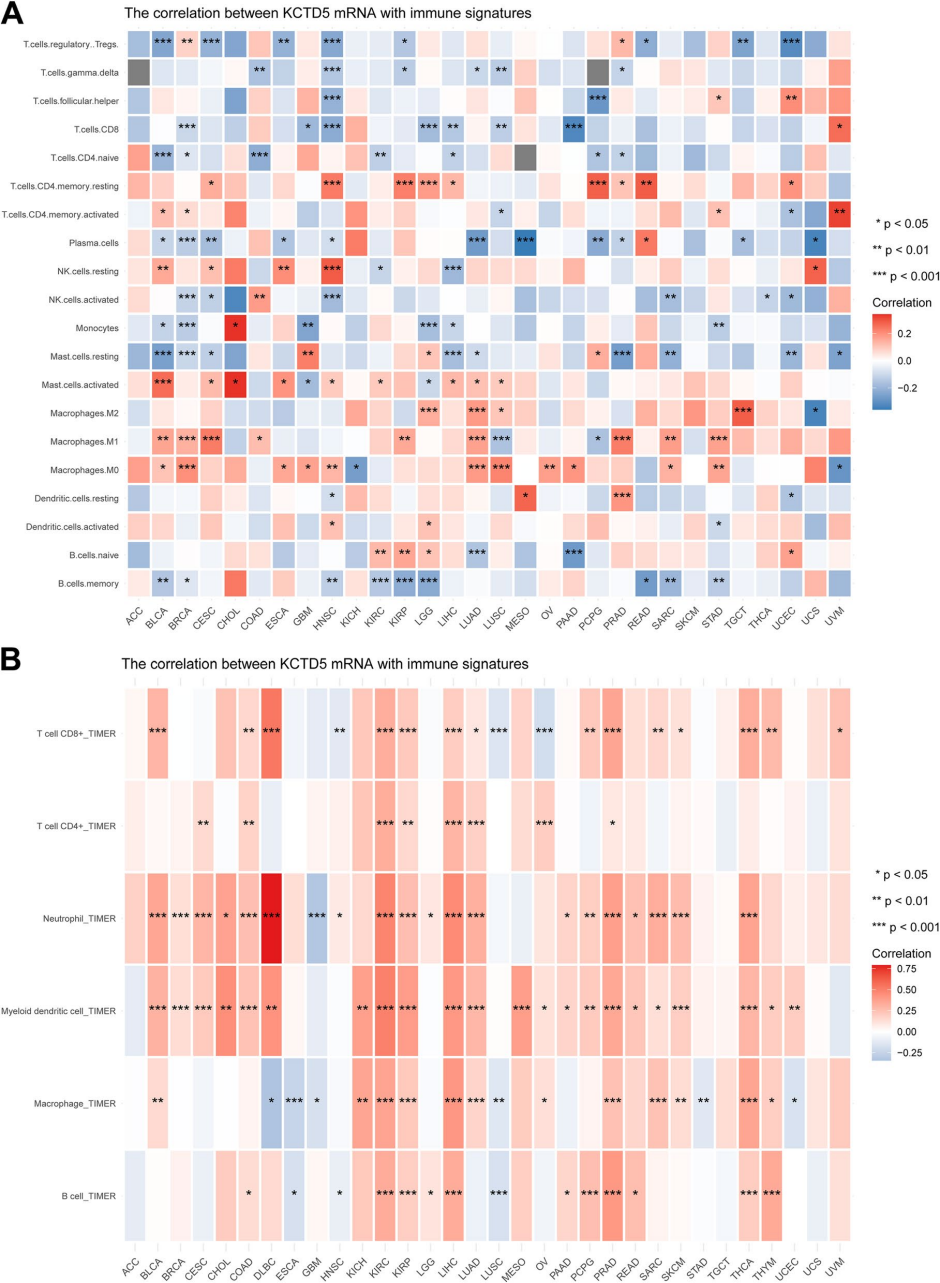
Correlations between KCTD5 expression and immune modulators in pan-cancer. **A** The correlation between the expression of KCTD5 and the infiltration levels of different immune cell subtypes. **B** The expression of KCTD5 was significantly associated with the abundance of infiltrating immune cells

The immune microenvironment plays an important role in the malignant progression of tumors, and immunomodulators related to the occurrence and development of tumors may serve as potential new targets for tumor therapy. In this study, the R package “UCSCXenaShiny” was used to analyze associations between KCTD5 gene expression and immune signatures/tumor immune cell infiltration in the TCGA database. Figure 5A shows the correlations between KCTD5 expression and the level of infiltration by various immune cell subtypes; KCTD5 was positively correlated with M0 macrophages, memory resting CD4 + T cells and M1 macrophages in various tumors but negatively correlated with plasma cells, resting mast cells and memory B cells. Figure 5B shows significant correlations between KCTD5 expression and the abundance of infiltrating immune cells: significant correlations with CD8 + T cells were found in 17 types of cancer, with CD4 + T cells in 8 types of cancer, with neutrophils in 20 types of cancer, with DCs in 22 types of cancer, with macrophages in 18 types of cancer, and with B cells in 14 types of cancer.

**Fig. 6:**
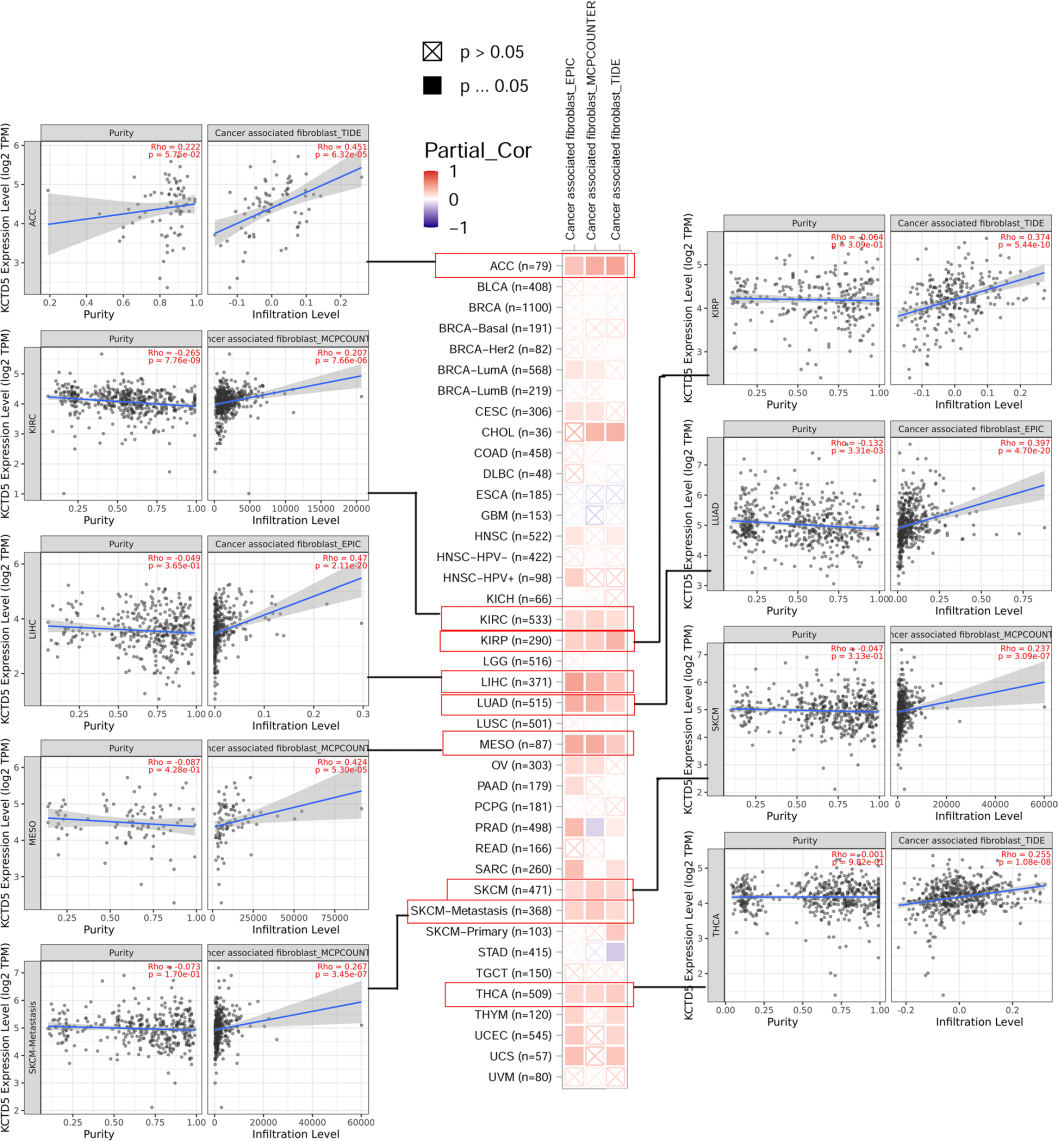
Correlation analysis between KCTD5 expression and immune infiltration of cancer-associated fibroblasts (CAFs) in pan-cancer

As the main components of the tumor microenvironment (TME), cancer-associated fibroblasts (CAFs) play an important role in the development and metastasis of tumors. We analyzed the association between KCTD5 expression and the level of infiltration by CAFs in pan-cancer (Fig. 6). Through the EPIC, MCPCOUNTER and TIDE algorithms, it was found that KCTD5 expression is positively correlated with infiltration by CAFs in ACC (Rho = 0.451, *P* = 6.32e-05), KIRC (Rho = 0.207, *P* = 7.66e-06), KIRP (Rho = 0.374, *P* = 5.44e-10), LIHC (Rho = 0.47, *P* = 2.11e-20), LUAD (Rho = 0.397, *P* = 4.70e-20), MESO (Rho = 0.424, *P* = 5.30e-05), SKCM (Rho = 0.237, *P* = 3.09e-07), SKCM-metastasis (Rho = 0.267, *P* = 3.45e-07) and THCA (Rho = 0.255, *P* = 1.08e-08) (Fig. 6).

**Fig. 7:**
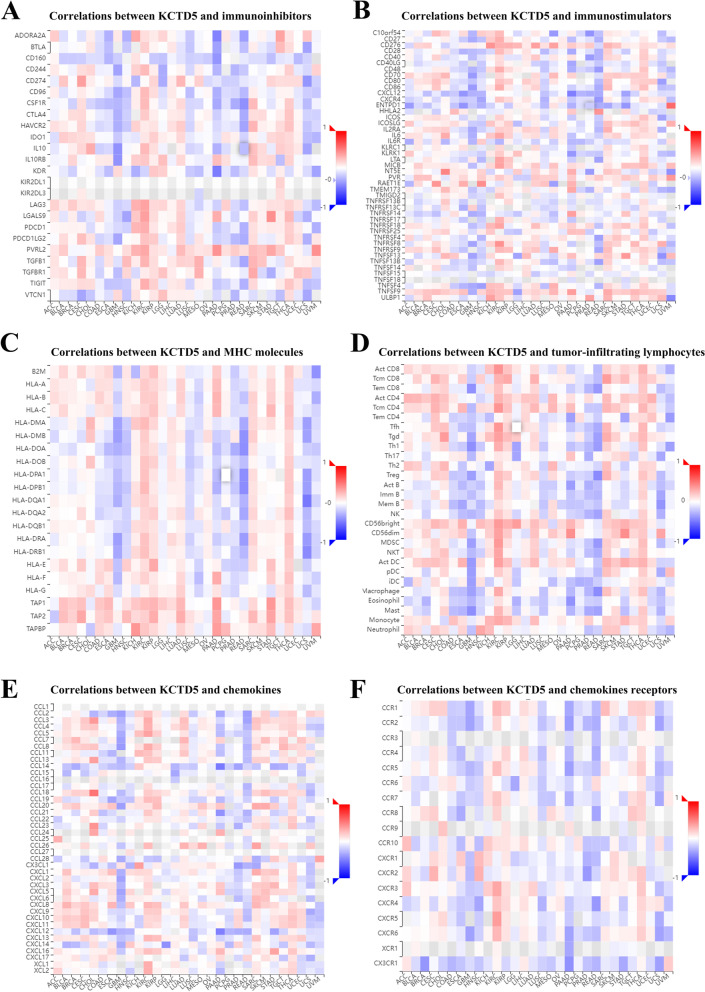
Correlation of KCTD5 and immunoregulation-related genes in pan-cancer. Correlations between KCTD5 expression and (**A**) Immunoinhibitors, **B** Immunostimulators, **C** MHC molecules, **D** Tumor-infiltrating lymphocytes (TILs), **E** Chemokines, **F** Chemokines receptors

Subsequently, we performed a co-expression analysis on KCTD5 and immune-related genes (genes that encode or are related to immunoinhibitors, immunostimulators, MHC molecules, tumor-infiltrating lymphocytes, chemokines, and chemokines receptors) in pan-cancer. The majority of immune-related genes in KIRC, KIRP, LUAD, SARC, and THCA showed a positive correlation with KCTD5, while in GBM, READ and UCS there were negative correlations (Fig. 7).

### Functional analysis based on KCTD5 expression

**Fig. 8:**
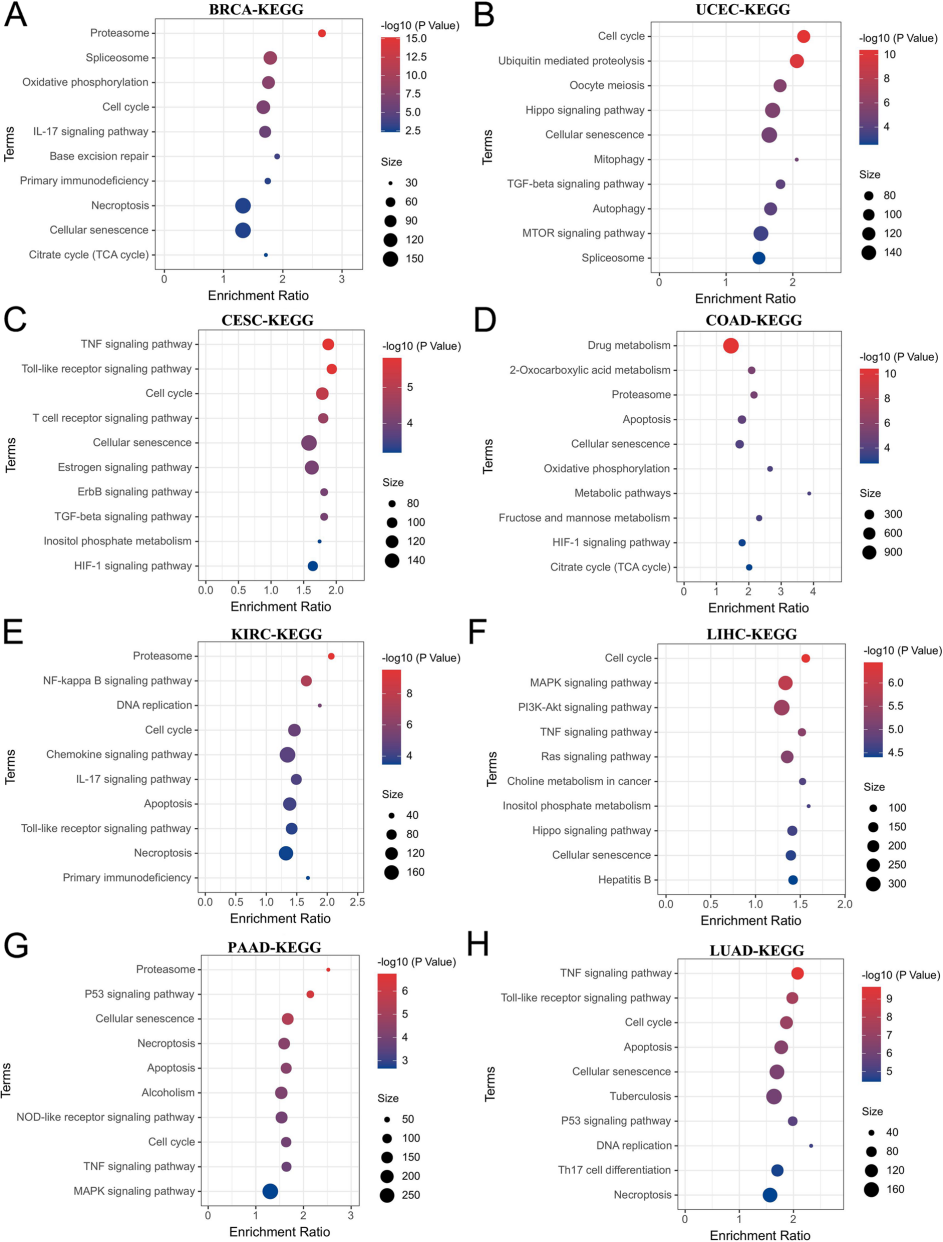
The signaling pathway analysis of KCTD5 in human cancers. **A** BRCA. **B** UCEC. **C** CESC. **D** COAD. **E** KIRC. **F** LIHC. **G** PAAD. **H** LUAD

**Fig. 9:**
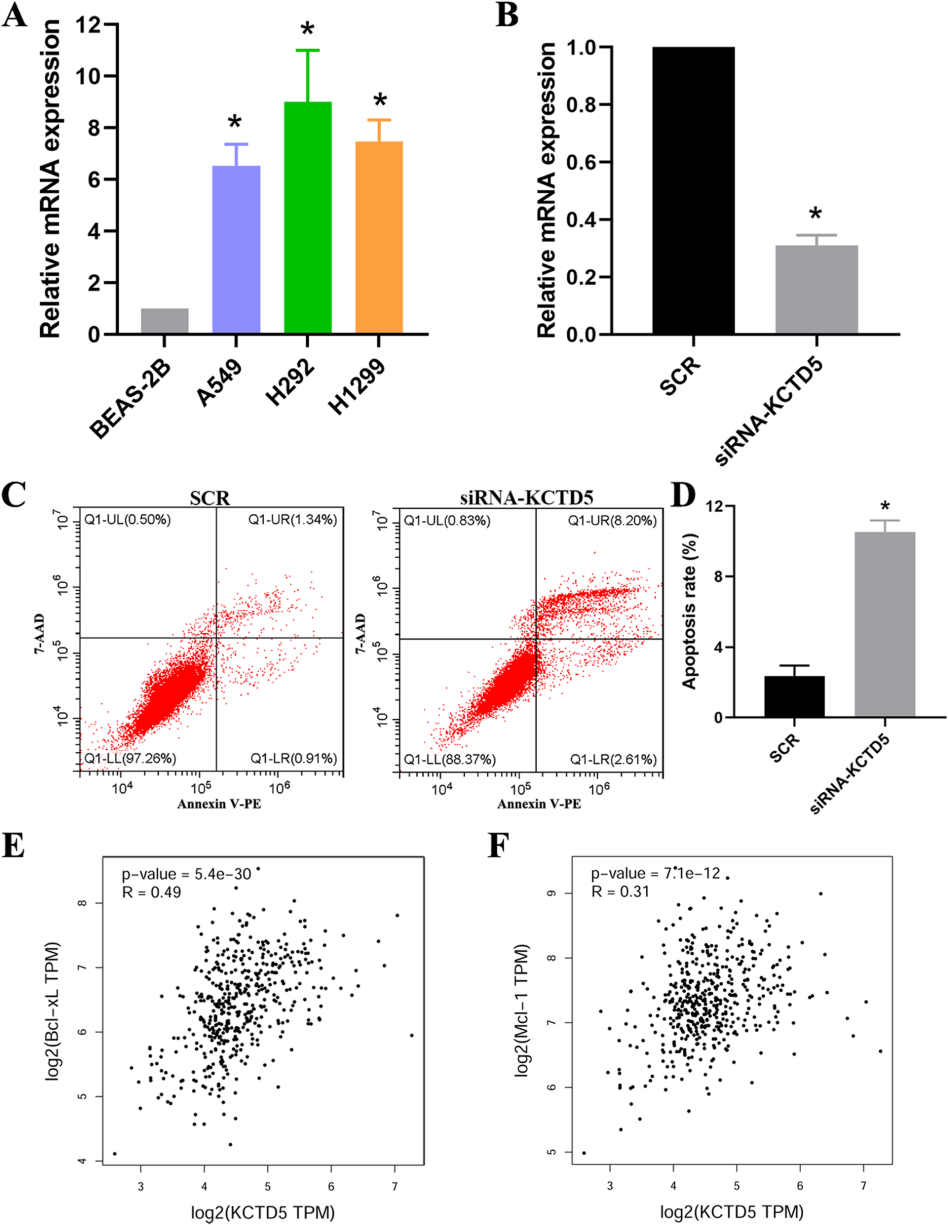
KCTD5 inhibited cell apoptosis in LUAD. **A** The mRNA expression of KCTD5 in lung cancer cells. **B** The siRNA effectively regulated the expression of KCTD5 in the A549 cell line. SCR, scrambled negative control; si, small interfering. **C**, **D** In A549, the apoptosis rate was significantly increased in the KCTD5 silenced group (siRNA-KCTD5) relative to the control group (SCR). **E**, **F** Correlation analysis revealed that KCTD5 was positively correlated with anti-apoptotic genes Bcl-xL and Mcl-1

To elucidate the molecular function of KCTD5 in tumors, we conducted KEGG enrichment analysis using the LinkedOmics database. We found that high expression of KCTD5 was significantly associated with proteasome (*P* = 6.66e-15), spliceosome (*P* = 1.13e-09), oxidative phosphorylation (*P* = 2.53e-08), cell cycle (*P* = 1.33e-07), IL-17 signaling pathway (*P* = 1.74e-06), base excision repair (*P* = 2.04e-04), primary immunodeficiency (*P* < 0.05), necroptosis (*P* < 0.05), cellular senescence (*P* < 0.05), and citrate cycle (*P* < 0.05) in BRCA (Fig. 8A); with the cell cycle (*P* = 4.18e-11), ubiquitin mediated proteolysis (*P* = 1.48e-10), oocyte meiosis (*P* = 2.44e-06), hippo signaling pathway (*P* = 4.19e-06), autophagy (*P* = 7.64e-05), and mTOR signaling pathway (*P* = 4.89e-04) in UCEC (Fig. 8B); with the TNF signaling pathway (*P* = 1.51e-06), cell cycle (*P* = 6.54e-06), and HIF-1 signaling pathway (*P* = 6.52e-04) in CESC (Fig. 8C); with drug metabolism (*P* = 3.72e-11), apoptosis (*P* = 3.32e-05), metabolic pathways (*P* = 1.40e-04), HIF-1 signaling pathway (*P* < 0.05), and citrate cycle (*P* < 0.05) in COAD (Fig. 8D); with the cell cycle (*P* = 9.76e-06), apoptosis (*P* = 8.09e-05), and necroptosis (*P* = 3.03e-04) in KIRC (Fig. 8E); with the cell cycle (*P* = 3.64e-07), MAPK signaling pathway (*P* = 1.21e-06), and PI3K-Akt signaling pathway (*P* = 3.16e-06) in LIHC (Fig. 8F); with necroptosis (*P* = 4.38e-05), apoptosis (*P* = 4.63e-05), and cell cycle (*P* = 1.24e-04) in PAAD (Fig. 8G); with the TNF signaling pathway (*P* = 2.16e-10), cell cycle (*P* = 6.87e-08), apoptosis (*P* = 2.81e-07), and necroptosis (*P* = 3.56e-05) in LUAD (Fig. 8H). Interestingly, KCTD5 was found to be associated with apoptosis, necroptosis and autophagy. We also examined the level of apoptosis in lung cancer cell lines and the association between KCTD5 and apoptosis-related genes. First, we measured the mRNA expression of KCTD5 in BEAS-2B, A549, H292, and H1299. The results indicated that expression of KCTD5 was significantly higher in A549, H292, and H1299 than in BEAS-2B, consistent with the results shown in Fig. 1 (Fig. 9A). In addition, we measured apoptosis in A549 cells. In those cells, the apoptosis rate was significantly increased in the KCTD5-silenced group (siRNA-KCTD5) relative to the control group (SCR) (Fig. 9B, C, D). Correlation analysis revealed that KCTD5 was positively correlated with anti-apoptotic genes Bcl-xL (*P* = 5.4e-30, R = 0.49) and Mcl-1(*P* = 7.1e-12, R = 0.31) (Fig. 9E, F). These results suggest that KCTD5 is involved in regulating the development of various tumors and that it may inhibit tumor cell apoptosis in LUAD.

### Drug sensitivity analysis based on KCTD5 expression

**Fig. 10:**
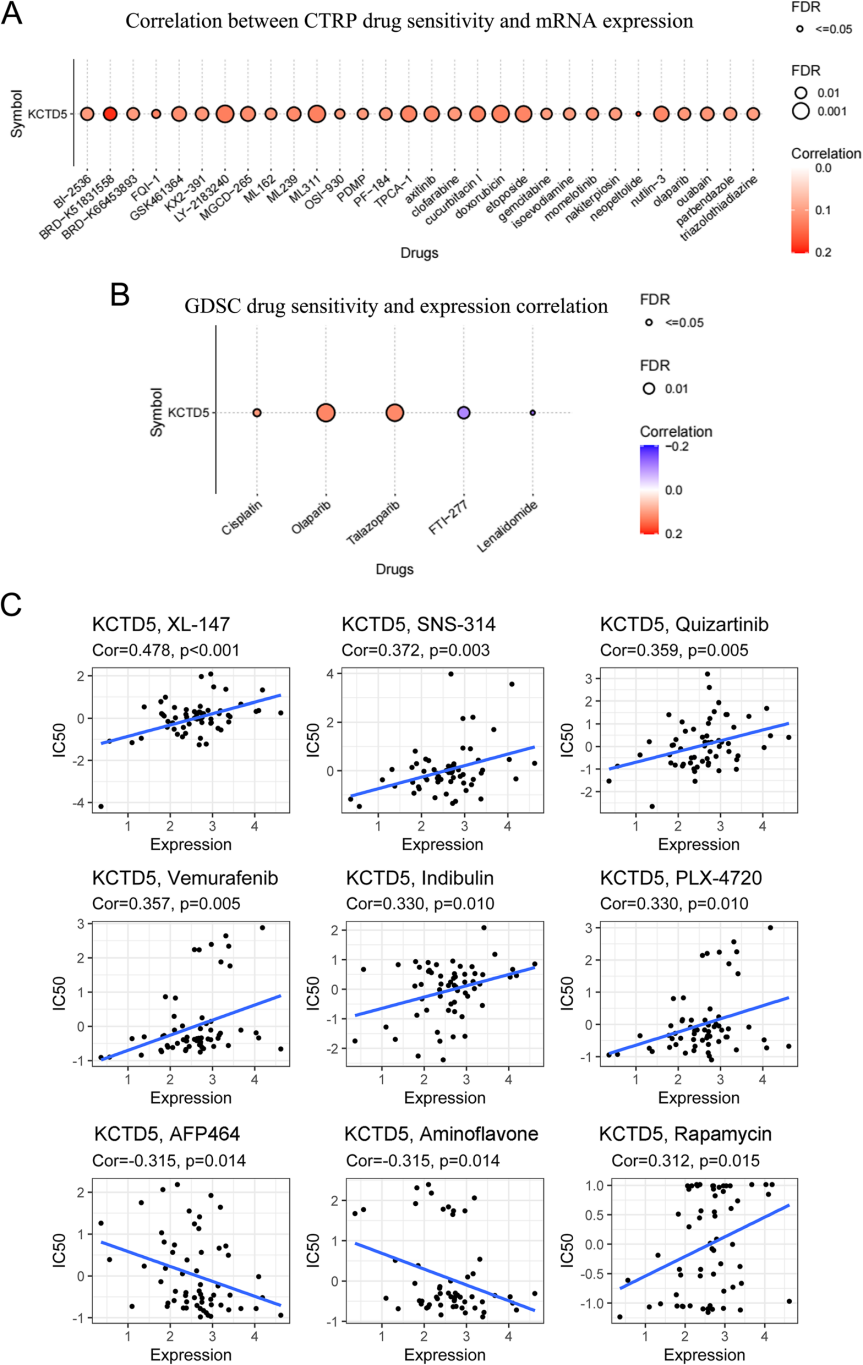
Drug sensitivity analysis based on KCTD5 expression using the three different databases CTRP (**A**), GDSC (**B**), Cellminer (**C**). *P* < 0.05 was considered statistically significant

We investigated a potential correlation between drug sensitivity and KCTD5 expression using three different databases (CTRP, GDSC, and Cellminer). According to the CTRP database, doxorubicin, cucurbitacin I, TPCA-1, nutlin-3, axitinib, MGCD-265, momelotinib, olaparib, OSI-930, and gemcitabine were the top ten drugs that showed positive correlations with KCTD5 expression (Fig. 10A). The GDSC database showed that KCTD5 expression was positively correlated with sensitivity to olaparib, talazoparib, and cisplatin; in contrast, it was negatively correlated with sensitivity to FTI-277 and lenalidomide (Fig. 10B). Using the Cellminer database, we correlated KCTD5 expression with sensitivity to 263 FDA-approved drugs in 60 tumor cell lines; the nine drugs with the strongest correlations with KCTD5 expression were XL-147, SNS-314, quizartinib, vemurafenib, indibulin, PLX-4720, AFP464, aminoflavone, and rapamycin (Fig. 10C). These results show that KCTD5 expression may serve as a predictor of the response of tumors to chemotherapeutic drug treatment.

## Discussion

Previous studies have shown that KCTD5 is associated with tumor development. For example, Rivas et al. reported that KCTD5 positively regulates TRPM4 activity by enhancing Ca^2+^ sensitivity and that KCTD5 promotes cell migration and contractility in breast cancer cells by affecting TRPM4 [20]. Canales et al. showed that KCTD5 regulates the proliferation and adhesion dynamics of murine melanoma cells through its effects on Rac1 activity and the Ca^2+^-signaling pathway, thereby acting as a regulator of cell migration [21]. Pan-cancer analysis of KCTD5 from the perspective of the tumor as a whole has not been reported in the literature. Pan-cancer analysis can be used to identify similarities and differences among different tumors, promote the screening of new therapeutic targets and provide new insights related to tumor prevention and treatment. In the present study, we comprehensively analyzed KCTD5 gene across multiple databases as a basis for evaluating the molecular function of KCTD5 in pan-cancer and identifying its value in tumor prognostic prediction and drug therapy.

The tumor immune microenvironment is an important regulator of tumor development, invasion and metastasis and is mainly composed of infiltrating immune cells, chemokines and cytokines [22, 23]. We performed immunoinfiltration analysis of common types of immune cells (CD4 + T cells, CD8 + T cells, neutrophils, macrophages, B cells and dendritic cells). The results showed that expression of KCTD5 was significantly correlated with the abundance of infiltrating immune cells. Tumor-associated fibroblasts are important components of the tumor microenvironment. Tumor-associated fibroblasts secrete growth factors, chemokines, and proteases that regulate the functions of innate and adaptive immune cells [24, 25]. Through various algorithms, we found that KCTD5 expression is positively correlated with infiltration by CAFs in ACC, KIRC, KIRP, LIHC, LUAD, MESO, SKCM, SKCM metastasis and THCA. The immune landscape in pan-cancer suggests that KCTD5 plays an important role in regulating the tumor immune microenvironment.

Accumulating evidence has revealed that escape from cell death and abnormal proliferation are the most important features of tumorigenesis [26]. Therefore, inhibiting the proliferation of tumor cells and inducing their death is a key step in cancer treatment. Currently known types of programmed cell death mainly include apoptosis, necroptosis, autophagy, ferroptosis, pyroptosis, and cuproptosis. Interestingly, KCTD5 was found to be associated with apoptosis, necroptosis and autophagy in LUAD, PAAD, KIRC, BRCA, COAD, and UCEC. Moreover, in vitro experiments further confirmed that overexpression of KCTD5 inhibits apoptosis in LUAD. Correlation analysis revealed that KCTD5 is positively correlated with expression of the anti-apoptotic genes Bcl-xL and Mcl-1. Our results preliminarily suggest that KCTD5 is involved in the regulation of tumor cell apoptosis, but the specific regulatory mechanism remains unclear. Regulation of the apoptosis signaling pathway is an important way to improve cancer therapy [27, 28]. KCTD5 may be a potential target for tumor therapy.

Drug resistance is an important cause of death in cancer patients [29]. In recent years, with the application of genome-wide screening methods such as deep sequencing and single cell sequencing, the response of cancer to treatment and the evolution of treatment after exposure have been revealed, and new insights have been provided for reversing drug resistance and improving therapeutic effect [30, 31]. We investigated a potential correlation between drug sensitivity and KCTD5 expression using CTRP, GDSC, and Cellminer databases. The results suggest that the expression of KCTD5 is positively correlated with the sensitivity of many classical antitumor drugs, such as cisplatin, gemcitabine, and doxorubicin. KCTD5 may be used as a predictor of tumor response to chemotherapy.

There are some limitations in this study. We have extensively discussed the molecular function of KCTD5 in pan-cancer, but there is a lack of clinical validation. Based on public database screening, we found that the expression of KCTD5 is associated with the sensitivity of antitumor drugs, further experimental verification needed. In addition, the specific molecular mechanism by which KCTD5 regulates programmed cell death remains unclear. In the future, we will further explore the key apoptotic signaling pathways and targets in the regulation of tumor apoptosis by KCTD5, providing a new theoretical basis for effective tumor therapy.

In conclusion, our first pan-cancer study of KCTD5 demonstrated that this gene is highly expressed in most cancers and revealed an association between KCTD5 expression and CNV, SNV, and DNA methylation. Survival analysis suggested that KCTD5 can be used as a prognostic factor for various tumors. Furthermore, KCTD5 was significantly associated with the tumor immune microenvironment, programmed cell death, and drug sensitivity. The findings may help elucidate the role of KCTD5 in tumorigenesis as well as offer new insights for tumor precision therapy.

## Supplementary Information

**Additional file 1: Supplementary Figure 1.** The protein expression of KCTD5 in pan-cancer (UALCAN). *P < 0.05, ***P < 0.001.

## Data Availability

The datasets generated and/or analysed during the current study are available in the Cancer Genome Atlas (TCGA) database, UCSC Xena (https://xena.ucsc.edu/), the GEPIA2 database (http://gepia2.cancer-pku.cn/), the HPA database (https://www.proteinatlas.org/), the UALCAN database (https://ualcan.path.uab.edu/), the TISIDB database (http://cis.hku.hk/TISIDB/), Kaplan–Meier Plotter, PrognoScan (http://dna00.bio.kyutech.ac.jp/PrognoScan/index.html), the GSCA database (http://bioinfo.life.hust.edu.cn/GSCA/#/), the CellMiner database (https://discover.nci.nih.gov/cellminer/), the TIMER2.0 (http://timer.cistrome.org/), and the LinkedOmics database (http://linkedomics.org/login.php).
