# Supplementary material for: Identifies KCTD5 as a novel cancer biomarker associated with programmed cell death and chemotherapy drug sensitivity
Source: BMC Cancer. 2023 May 6;23:408. doi: 10.1186/s12885-023-10895-2 (PMC10163697; doi:10.1186/s12885-023-10895-2)
Supplement: Supplementary file 1 — Additional file 1: Supplementary Figure 1. The protein expression of KCTD5 in pan-cancer (UALCAN). *P < 0.05, ***P < 0.001. [file 12885_2023_10895_MOESM1_ESM.docx]

**Identifies KCTD5 as a novel cancer biomarker associated with programmed cell death and chemotherapy drug sensitivity**

Yuan-Xiang Shi^1,^ *, Jian-Hua Yan^2^, Wen Liu^3^, Jun Deng^3^

^1^Institute of Clinical Medicine, Hunan Provincial People’s Hospital, The First Affiliated Hospital of Hunan Normal University, Changsha, Hunan 410005, P.R. China;

^2^Department of Cardiac Thoracic Surgery, Hunan Provincial People’s Hospital, The First Affiliated Hospital of Hunan Normal University, Changsha, Hunan, China;

^3^Department of Pharmacy, Hunan Provincial People’s Hospital, The First Affiliated Hospital of Hunan Normal University, Changsha, Hunan 410005, P.R. China.

Correspondence to: Dr. Yuan-Xiang Shi, Institute of Clinical Medicine, Hunan Provincial People’s Hospital, The First Affiliated Hospital of Hunan Normal University, Changsha, Hunan 410005, P.R. China. E-mail: [yuanxiangshi@hunnu.edu.cn](mailto:yuanxiangshi@hunnu.edu.cn)

Supplementary materials


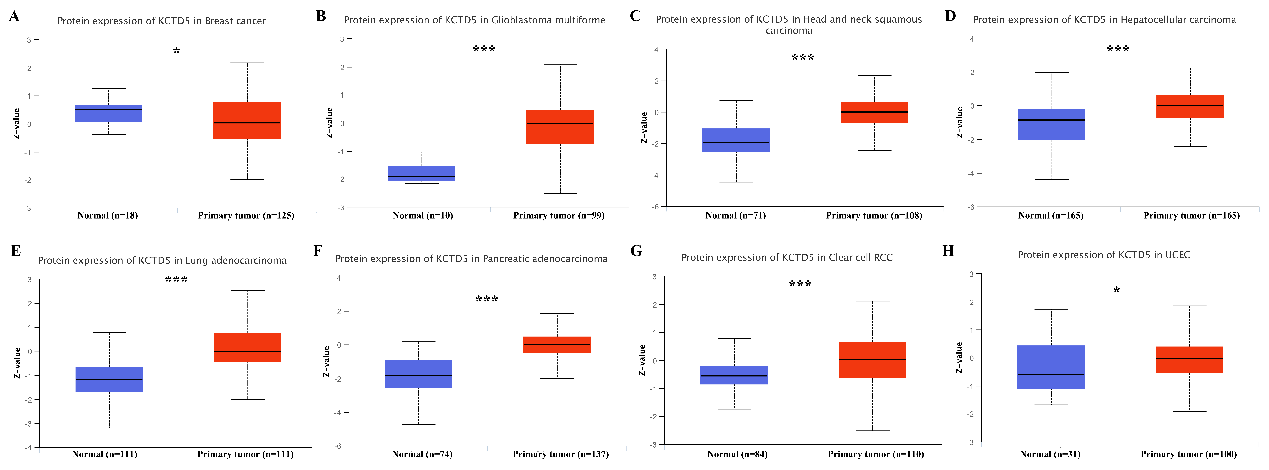


Supplementary Figure 1 The protein expression of KCTD5 in pan-cancer (UALCAN). *P < 0.05, ***P < 0.001.
